# Sensory mechanotransduction at membrane-matrix interfaces

**DOI:** 10.1007/s00424-014-1563-6

**Published:** 2014-07-02

**Authors:** Kate Poole, Mirko Moroni, Gary R. Lewin

**Affiliations:** Department of Neuroscience, Max-Delbrück Center for Molecular Medicine, Robert-Rössle Straße 10, D-13092 Berlin, Germany

**Keywords:** Mechanotransduction, Touch, Ion channels, Extracellular matrix, STOML3, Laminin-332

## Abstract

Sensory cells specialized to detect extremely small mechanical changes are common to the auditory and somatosensory systems. It is widely accepted that mechanosensitive channels form the core of the mechanoelectrical transduction in hair cells as well as the somatic sensory neurons that underlie the sense of touch and mechanical pain. Here, we will review how the activation of such channels can be measured in a meaningful physiological context. In particular, we will discuss the idea that mechanosensitive channels normally occur in transmembrane complexes that are anchored to extracellular matrix components (ECM) both in vitro and in vivo. One component of such complexes in sensory neurons is the integral membrane scaffold protein STOML3 which is a robust physiological regulator of native mechanosensitive currents. In order to better characterize such channels in transmembrane complexes, we developed a new electrophysiological method that enables the quantification of mechanosensitive current amplitude and kinetics when activated by a defined matrix movement in cultured cells. The results of such studies strongly support the idea that ion channels in transmembrane complexes are highly tuned to detect movement of the cell membrane in relation to the ECM.

## Introduction

The ability of cells to rapidly transform mechanical deflection or changes in applied force into an electrical signal is a property that is still poorly understood at the molecular level. It is probable that almost all cells sense and respond to mechanical changes in their vicinity. In this review, we will concentrate on fast mechanotransduction, a process by which many cells, especially sensory cells, rapidly transform mechanical stimuli into graded electrical signals. This process is often called mechanoelectrical transduction (MET) and likely requires that a mechanical stimulus directly gates an ion channel. Fast mechanotransduction entails the transformation of mechanical energy into an electrical signal in a sub-millisecond time frame. The gating of ion channels by mechanical stimuli has been studied for many years, and the molecular identity of the first truly stretch-sensitive ion channel was first identified in bacteria in the early 90s by Kung and his colleagues. This bacterial channel called mechanosensitive channel of large conductance (MscL) can be gated directly by membrane stretch without the need for any accessory proteins [93].

Elegant biophysical work over the last few decades has shown that the detection of sound waves by hair cells of the inner ear is an exquisitely sensitive process in which MET is critically dependent on the placement and mechanical coupling of the transduction channels [8, 37]. Thus, functional MET channels are placed at the tips of the hair cell stereocilia and require an intact tip-link connector between stereocilia in order to gate in response to bundle displacements of a few tens of nanometers [3, 34]. The molecular composition of the tip link is probably a combination of cadherin-23 and protocadherin-15 dimers which span the 150–200-nm distance between stereocilia tips [55, 90, 91]. Importantly, the presence of cadherin-23 or protocadherin-15 or intact tip links appear to be absolute requirements for hair cell mechanotransduction [3, 99]. In another well-studied example of a mechanoreceptor, the body touch neurons of the nematode *Caenorhabditis elegans* and the genetic and electrophysiological studies have definitively demonstrated that the Deg/ENaC family members MEC-4 and MEC-10 form the core of a mechanosensitive complex in these neurons [2, 33, 82, 83]. However, as for the hair cell, there appears to be a requirement for the presence of extracellular matrix molecules at least for the assembly of a sensitive mechanoreceptor response [32, 82]. The vertebrate hair cell and body touch receptor neurons from *C. elegans* both appear to be examples in which the mechanotransduction channel functions in the context of its associated proteins, including extracellular proteins.

The above examples serve to highlight an apparent dichotomy in which some mechanosensitive channels are directly gated by membrane stretch, and others have only been shown to be mechanosensitive in the presence of other proteins that may be necessary to couple mechanical energy to the ion channels in the membrane (see below). The detection of membrane stretch is undoubtedly an important function of mechanosensing ion channels. However, here, we will argue and provide evidence for the view that in many cases, physiologically relevant fast mechanotransduction, is mediated by transmembrane complexes localized to plasma membrane-matrix interfaces.

## The somatic mechanosensory system

One important system in which mechanotransduction occurs at membrane-matrix interfaces is the somatic sensory system. The largest and most diverse mechanosensing system of the vertebrate body is elaborated by somatic sensory neurons of the trigeminal and dorsal root ganglia. Almost all the organs of the body are innervated by one or more of these ganglia, and many, if not most, of these sensory neurons are responsive to mechanical stimuli. In a marked contrast to non-neuronal mechanosensory cells of the inner ear, these sensory neurons typically have an extremely long axon extending to the peripheral organ where branches are elaborated, forming endings that detect mechanical stimuli [64, 65]. It is important to realize that in all cases, the only compartments of the sensory neuron responsive to mechanical stimuli in vivo are the terminal branched endings. This can easily be demonstrated in vivo as mechanical stimulation of the nerve, ganglion or spinal cord does not initiate action potentials in sensory neurons [56]. However, if a peripheral nerve is cut or ligated, many axons acquire mechanosensitivity at the ligation site with a time course consistent with fast anterograde transport [56]. The strict localization of mechanosensitivity to the peripheral endings of sensory neurons suggests that there exist active mechanisms to accumulate, sequester and functionalize transduction molecules at the peripheral endings of the neuron. Indeed, we have previously provided evidence that specialized vesicle pools, called transducosomes, may be used to deliver transduction proteins to peripheral endings [61]. The morphology of sensory endings is complex, but can be divided into those neurons that form endings at specialized end organs like hair follicle afferents [64, 66, 67, 95], Merkel cell neurite complexes [50, 71, 97], Pacinian corpuscles [68, 95], Meissner’ corpuscles [45] and muscle spindles [4, 53], and those with so-called free nerve endings [57, 58]. The latter endings most often belong to sensory endings with nociceptive or thermoreceptive function. Nevertheless, sensory axons with free nerve endings are very often mechanosensitive, but only respond to higher-threshold mechanical stimuli. Morphological and ultrastructural studies have directly demonstrated that the sensory endings within specialized end organs are intimately connected to support cells within their end organ, be they terminal Schwann cells [66] or other cell types like Merkel cells [78, 79].

In the case of sensory afferents with free nerve endings, it is much harder to be sure where the likely sites of mechanosensory transduction reside within the terminal branches. However, even in this case, ultrastructural studies have demonstrated the existence of contacts between the membranes of the nociceptor axons and surrounding cells like keratinocytes [47, 57]. Thus, it is clear that in vivo mechanotransduction by sensory afferents takes place in the context of branched tubular endings that are in contact with neighbouring cells and may have proteinaceous connections with matrix within the end organ [42, 66]. The relevance of such cellular arrangements for the efficient and fast transduction of small mechanical stimuli relevant for touch perception will be discussed in this review. In particular, we will discuss the appropriateness of available methodologies for the direct recording of mechanoelectric transduction events for in vivo mechanotransduction.

## Methods for measuring the activity of mechanosensitive ion channels

The advent of the patch-clamp technique provided almost unlimited opportunities to directly measure the gating of ionic currents [88]. Thus, the ligand and voltage dependence of ion channel gating can easily be measured either in excised patches or in whole cells. Voltage can be controlled with extremely high precision using the classical voltage clamp paradigm, and thus, there is little ambiguity in determining the voltage dependence of ion channel gating. Similarly, ultra-fast methods for applying ligands to patches have allowed precise measurement of the kinetics with which ligand binding favours ion channel gating. In both cases, the precise control of the stimulation parameters is crucial for studying channel gating.

Patch-clamp methods have also been used to study how membrane stretch influences the gating of ion channels. Thus, mechanosensitive membrane currents were described soon after the invention of the patch-clamp technique. Initial studies in chick myotubes [41], Xenopus oocytes [76] and bacteria [5, 23, 72] revealed the presence of endogenous currents sensitive to the application of pressure to the membrane patch. These first experiments were conducted by establishing a seal between the recording pipette and the cell membrane, pressure was then applied directly to the solution in the recording pipette using a microprocessor-controlled piston connected to the electrode holder. This configuration allows the application of pressure exerted through the capillary walls to the membrane under the pipette [59]. The first systems had major drawbacks as they resulted in an uncertainty in the onset of the pressure pulse and a slow rising phase of the pulse itself, which precluded the analysis of the activation kinetics of channels with fast on and off kinetics [43, 74]. The technique was improved by Sachs and collaborators by the introduction of a piezoelectric bending element to control both pressure and vacuum, thus enabling the application of either positive or negative pressure to membrane patches [7]. This technical improvement increased the reliability and reproducibility of measurements of the kinetic properties of mechanically gated ion channels. Almost square wave steps of pressure can today be applied to membrane patches, and channel activity can be recorded in response to a pressure “jump”, similar to what was first established for ligand-gated ion channels [17, 35]. However, fast perfusion systems driven by piezo elements offer the possibility of solution exchange in the range of few hundred microseconds, pressure jumps with a high-speed pressure clamp are still limited by a slow rise and decay time in the range of a few milliseconds especially at high pressure values [7], thus precluding an accurate measure of the rise time of mechanically gated currents in sensory cells, which can activate with time constants of much less than 1 ms [14, 47, 48, 62].

The application of pressure steps via the patch pipette has allowed workers to study the properties of many mechanically gated currents in a variety of cultured cells. Pressure steps can be applied to patches in the cell-attached configuration where channels in the patch are presumably gated in an environment which still contains cortical cytoskeleton and where cytoplasmic factors can still influence gating. Uhtaek Oh and his colleagues successfully used this method to identify several distinct mechanosensitive currents in the membranes of cultured adult sensory neurons [15, 16]. Pressure steps can also be applied to excised patches containing channels either in “outside-out” or “inside-out” configurations; in some cases, the membrane bleb may still contain protein material from the cytoskeleton [87, 92]. However, in this latter case, the channel activity is measured in a context lacking mechanical elements linked to the cell cytoskeleton. The following considerations should be taken into account when characterizing mechanically gated channels using pressure jumps. First, since the pressure is applied via the patch pipette, the membrane exposed to the pressure step will typically only be a few square micrometres. Second, most such measurements have been made in cultured cells, and the nature of this technique requires that the recordings are made from patches of membrane acquired from the top of the cultured cell. Thus, access to channels that may be exclusively or predominantly present at membranes immediately adjacent to the cell culture substrate will be difficult, if not impossible, with such an approach. Of course, there are instances where a specialized area of membrane on the surface of cells rich in ion channels can be examined with such an approach. Thus, in the case of hair cells, the stereocilia bundle transduction channels are localized to the tips of just certain rows of stereocilia [8]. It has long been speculated that the cilia of endothelial cells may represent specialized mechanosensitive organelles that detect flow or shear stress as fluids flow across the cell, e.g. in a kidney tubular compartment [1, 54, 80]. Recently, directed patch recordings from membrane compartments containing cilia showed directly that these are enriched for mechanosensitive ion channels [22].

In order to overcome the low density of channels in many cell types, the “outside-out” or “macropatch” configuration has advantages, as the surface of membrane pulled from the cell is considerably larger than the one under the pipette in cell-attached configuration. However, in excised patches, not only are soluble cytoplasmic components lost, but more importantly, mechanically gated channels lose their connection to cytoplasmic proteins which could act as tethers or membrane scaffolds that are functional components of the MET complex. Using pressure steps applied in the on-cell or excised patch configuration, the pressure jump serves to stretch and simultaneously thin the plasma membrane being monitored electrophysiologically. Indeed, functional and X-ray crystallographic approaches have demonstrated that prokaryotic mechanosensitive channels are gated as the membrane thins; thus, membrane stretch leads to a pronounced tilting of alpha helixes within the channel that allows the pore to form [84]. Eukaryotic two-pore domain K^+^ channels (K2P) have long been known to be exquisitely sensitive to membrane stretch when measured in mammalian cells [13, 46, 69, 70]. Indeed, biophysical experiments indicated that the gating of these channels is modulated by the shape of lipids in the membrane. They activate in a range of 10 to 30 mmHg, and their gating is modulated by fatty acids such as arachidonic acid and membrane crenators [70]. Recently, two of the mammalian K2P channels, TREK1 and TRAAK, were shown by Mackinnon and colleagues to be directly gated by membrane stretch, positive and negative, after reconstitution into lipid bilayers without associated proteins [10]. However, another research group using a similar approach found that TREK1 was intrinsically mechanosensitive in lipid membranes, but was actually closed by the application of positive pressure [6]. It is not yet clear why these two research groups should find fundamentally different modes of mechanosensitivity for the same channel reconstituted in lipid membranes. The mammalian TRAAK and TREK channels are polymodal in nature in that they can be gated by temperature as well as membrane stretch. These channels are expressed in sensory neurons where they may be involved in setting the background K^+^ leak conductance that governs the resting membrane potential. Gene deletion studies have shown that these channels do regulate the excitability of mouse sensory neurons to natural stimuli like temperature and pressure [81]; however, it is unclear to what extent the specific mechanosensitivity of the K2P channel is responsible for such in vivo phenotypes. The prokaryotic MscL and MscS channels both exhibit dramatic lipid-induced conformational changes associated with gating [23, 93]. In patch-clamp experiments, MscL is activated at membrane tensions that are close to the lytic limit of the cell [93]; however, MscS is activated by lower tensions similar to those described for the gating of eukaryotic channels like TREK1 and TRAAK [10]. Thus, channel proteins directly gated by membrane stretch can have intrinsic molecular features that regulate their sensitivity to membrane tension.

Recently, two founding members, Piezo1 and Piezo2, of a new mechanosensitive channel family were discovered [19]. These two proteins are extremely large with more than 30 predicted membrane spanning segments, and they are apparently not related to any known ion channel family. Initially, Piezo1-dependent non-selective cation currents were found in neuroblastoma cells using cell indentation as a stimulus. Experiments using excised patches with pressure-clamp stimuli suggested that these channels also sense changes in the lipid bilayer tension [19, 39, 40]. Definitive proof that these proteins do form pore-forming channels was obtained by purifying the mouse Piezo1 protein and incorporating it into artificial bilayers where channel activity could be measured [20]. Interestingly, no evidence has been presented to date to suggest that purified and reconstituted Piezo1 channels are directly gated by membrane stretch [20]. The Piezo channels are readily gated by cell indentation and activate relatively rapidly but usually inactivate during steady-state indentation. Cell indentation is, however, a very imprecise and poorly controlled stimulus with which to quantify the kinetics of current activation and inactivation. Pressure-clamp experiments have shown that Piezo1 and Piezo2 both rapidly inactivate under conditions of constant pressure applied to excised patches [39, 40]. The Piezo2 protein has kinetics of activation and inactivation that are reminiscent of a rapidly adapting mechanosensitive current found predominantly in large-diameter dorsal root ganglion neurons [14, 18, 29, 31, 47, 48, 62, 63, 75], and initial knockdown studies have suggested that this rapidly adapting current is dependent on the presence of Piezo2 [19]. Cell indentation techniques have now been widely used to evoke mechanosensitive currents in sensory cells. Indeed, a non-selective cationic mechanosensitive current with kinetic properties and pharmacology similar to Piezo2 channels has recently been identified in Merkel cells [51, 71, 97]. Genetic ablation of Piezo2 in these cells abolished their mechanosensitivity to an indentation stimulus and attenuated the sustained firing of slowly adapting type I afferents that innervate the Merkel cell [71, 97].

## Matrix interactions relevant for fast mechanotransduction in sensory neurons

In contrast to studies on the sensory hair cell, it has so far proved impossible to make direct high-resolution intracellular recordings from mammalian afferent endings near the site of mechanosensory transduction. The most direct electrophysiological recordings in vivo were made more than four decades ago from Pacinian corpuscle afferents or muscle spindle endings in the cat. This was possible because these mechanoreceptor axons are, in the cat, exceptionally large (circa 50 μm in diameter) which allowed the experimenters to make low noise DC recordings of the receptor potential when the end organ was bathed in drugs, like lidocaine, that block action potential initiation [53, 68]. In 1996, Cesare and McNaughton showed that acutely cultured sensory neurons possess a heat-gated cation conductance, which they termed *I*_heat_, that exhibits a threshold and response function matching that of heat-sensitive nociceptors [12]. Several groups looked for mechanosensitive currents in acutely cultured sensory neurons that might correspond to a native receptor current activated by physiologically relevant mechanical stimuli. In 1997, Cunningham and colleagues demonstrated that fluid jet stimulation of the neurites of putative aortic baroreceptor neurons could evoke inward currents measured with the whole-cell patch-clamp technique [21]. Later, several groups used direct mechanical stimulation of the cell body or neurites of a cultured rodent’s dorsal root ganglion neurons to show that rapidly activating mechanically gated currents can be readily measured in these cells [18, 31, 48, 75]. It is now generally agreed that the direct poking of the cell body can evoke both rapidly inactivating and slowly inactivating currents in the majority of cultured dorsal root ganglion (DRG) neurons. There is still, however, a lack of consensus on the basic properties of these currents, as measurements of threshold, activation time constants, inactivation time constants, ionic selectivity and pharmacological sensitivity can vary significantly between laboratories. Some of these discrepancies may be due to methodological issues stemming from variations in the way that mechanical stimuli are delivered to the cell. For example, labs using a classical piezo-driven motor to stimulate cultured neurons report surprisingly large indentations (often >5 μm) needed to evoke mechanosensitive currents, whereas labs using the Kleindiek nanomotor device to stimulate neurites or cells have seen currents evoked with much smaller stimuli (often <1 μm). It is likely that the mode of mechanical stimulation will be very difficult to standardize as this method is intrinsically inaccurate due to several factors, discussed in more detail below. One major factor is the fact that it is impossible to accurately determine the absolute magnitude of the stimulus when indenting the cell. Usually, a rounded polished glass micropipette is used to indent the cell, but the starting point for the stimulus is necessary at some unknown distance from the neuronal membrane. For example, if a stimulus of 1 μm is given and this leads to current activation, the experimenter can only guess that the starting point for the stimulation was a value between 0 and 1 μm. All groups who have examined mechanosensitive currents in cultured DRG neurons observe a diversity of mechanosensitive currents with different inactivation time constants (*τ*). Thus, mechanosensitive currents have been classified as rapidly adapting (RA, *τ* < 5 ms), intermediately inactivating (IA, *τ* 5–50 ms) and slowly adapting (SA, *τ* > 50 ms) [47, 48, 62, 63]. Although there are differences in the details of how groups classify currents according to their inactivation properties, e.g. see Delmas [24], there is broad agreement about the existence of rapidly and slowly inactivating currents. There is also agreement about the finding that RA currents are found in both mechanoreceptors and nociceptors whereas SA currents are found more or less exclusively in nociceptors [25, 30, 31, 48, 62, 63].

There has been surprisingly little work done on the pharmacology of distinct mechanosensitive currents in sensory neurons. In our hands, using a nanomotor to mechanically stimulate sensory neuron neurites in culture, Ruthenium red was not effective in blocking the RA current, but did reversibly block the SA-type current [48]. Using a piezo-driven motor, Drew and colleagues have recorded RA currents with slightly slower kinetics than those that we have observed and found a reversible block of this current with Ruthenium red. Sensory neuron mechanosensitive currents have been found by two groups to be completely insensitive to block by amiloride and its analogues like benzamil [31, 48]. However, there is one report that amiloride blocks a considerable portion of the mechanosensitive current in both putative mechanoreceptors and nociceptors isolated from rats [18]. Reversibility of the amiloride block was, however, not demonstrated. There is broad agreement that in sensory neurons, slowly inactivating mechanosensitive currents are non-selective cation currents that are susceptible to block by both gadolinium ions and Ruthenium red. However, we have consistently observed very sensitive and fast RA currents that are insensitive to Ruthenium red, reverse at positive potentials and are sodium selective [14, 47, 48, 62, 85]. The mode of mechanical stimulation is undoubtedly important, and it is clear that there is a large variation in the speed of the mechanical indentation used as well as its magnitude; speeds of stimulation vary from 200 to 7,500 μm/s [18, 47]. The speed is probably a very relevant factor especially considering the fact that the activation of the RA-mechanosensitive current is critically dependent on the stimulus velocity [44, 86].

**Fig. 1:**
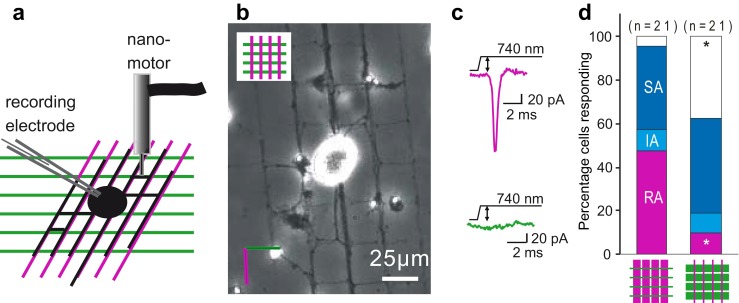
Local inhibition of mechanically gated currents by laminin-332. **a** Schema of experimental setup to test the local effects of substrate composition on mechanically gated currents. Somatosensory neurons were acutely prepared from mice and cultured on cross-hatched patterns of laminins created using microcontact printing; *magenta* indicates EHS-laminin and *green* EHS-laminin/laminin-332, 15:1. Cells were monitored using whole-cell patch clamp, and a nanomotor was used to indent neurite segments of the same cell over the different substrates. **b** When sensory neurons are cultured on such patterns, neurites grow exclusively over the printed regions. **c** Representative current traces of the cellular response when a cell is probed over the EHS-laminin substrate (*magenta trace*) vs over the EHS-laminin/laminin-332, 15:1 substrate (*green trace*). **d** In matched measurements, the rapidly adapting (RA) current measured in a neurite segment over EHS-laminin was not observed in a significant number of cells when the same cell was stimulated at a neurite segment attached to the EHS-laminin/laminin-332, 15:1 substrate (Student’s *t* test, *p < 0.05). Data from [14]

Very fine mechanical stimuli can be delivered to cells or neurites, very rapidly with extremely small step sizes (10 nm or less) using a nanomotor device (Kleindiek, Nanotechnik) [48]. We found that mechanosensitive currents could be evoked more reliably and with smaller stimuli from the newly grown neurites of sensory neurons compared to their cell bodies [48]. This observation suggested that either mechanosensitive channels are enriched in neurite membranes or that they are more readily activated by the mechanical indentation which is in the latter case closer to the culture substrate. We thus hypothesized that the relevant mechanosensitive channels in sensory neurons may be preferentially activated at the membrane-matrix interface of sensory neurons. Indeed, using a combination of electron microscopy, biochemistry and electrophysiology, we were able to demonstrate the presence of a 100-nm long membrane tether at the sensory neuron matrix interface, the presence of which appears to be necessary for RA-current activation. Thus, short-term treatment with specific and non-specific proteases abolished our ability to measure RA-current activation, concurrently with the loss of the 100-nm tether [47]. Twenty four hours after protease treatment, we observed a coincident reappearance of both the tether and the RA-mechanosensitive current. Additionally, cultured sympathetic neurons that lack mechanosensitive currents also lack the tether. The tether protein we observed appears to bind at one end to the laminin-containing matrix, which is most often derived from Engelbreth-Holm-Swarm murine sarcoma cells (EHS-laminin). Interestingly, the subunit composition of the laminin substrate exerts powerful and local control over the presence of mechanosensitive currents and the tether protein. Thus, laminin-332, a trimeric molecule composed of the α3, β3 and γ2 laminin chains [28], which is present at the dermal-epidermal border of the skin, was found not to support RA currents in cultured primary sensory neurons [14]. It is known that EHS-laminin contains laminin-111 (α1, β1 and γ1 chains [52]), but EHS-laminin does not support RA-mechanosensitive currents when even small amounts of laminin-332 are present (a ratio of 15:1 of EHS-laminin to laminin-332 is sufficient). Thus, laminin-332 is profoundly inhibitory for the RA-mechanosensitive current, and we could show that this effect was contact-dependent and highly local in nature (Fig. 1). To demonstrate the localized nature of laminin-332-mediated inhibition of mechanosensitive currents, we used microcontact printing to prepare substrates where stripes of EHS-laminin (permissive for mechanotransduction channel gating) and stripes of EHS-laminin/laminin-332, 15:1 (non-permissive), were stamped onto a glass substrate in a cross-hatched pattern. Sensory neurons cultured on these patterned substrates extend neurites along laminin stripes, and we applied localized mechanical stimuli to neurite segments from the same neuron growing on laminin that is permissive or non-permissive for mechanosensitive currents (Fig. 1). Interestingly, RA-mechanosensitive currents were only evoked from neurite segments on EHS-laminin but were almost absent in neurites from the same cell in contact with a laminin-332-containing matrix. The tether protein we have identified appears to play an integral role as it was virtually absent from the neurite-matrix interfaces when laminin-332 was present [14]. These data suggest that extracellular matrix molecules, including the protease-sensitive tether we have identified, may form an integral part of the mechanotransduction complex. It is conceivable that, like hair cell mechanotransduction channels, sensory transduction channels are opened by mechanical energy that is transferred to the channel via mechanical elements that are linked to the extracellular matrix. In this context, it is interesting to note that somatic sensation and hearing share common genetic factors including genes encoding large extracellular proteins [36]. In our experiments, the influence of extracellular matrix and presence of a tether were most marked on the RA-mechanosensitive current [14, 47]. However, it is important to realize that SA-mechanosensitive currents that maybe distinct from the RA-mechanosensitive currents [48] were also profoundly modulated by the same factors. Thus, the experimental ablation of the tether or exposure of the neurons to laminin-332 matrix were both associated with a very marked slowing in the kinetics of SA-mechanosensitive currents manifested as a very marked slowing of activation kinetics as well as very prolonged latencies for current activation [14, 47].

**Fig. 2:**
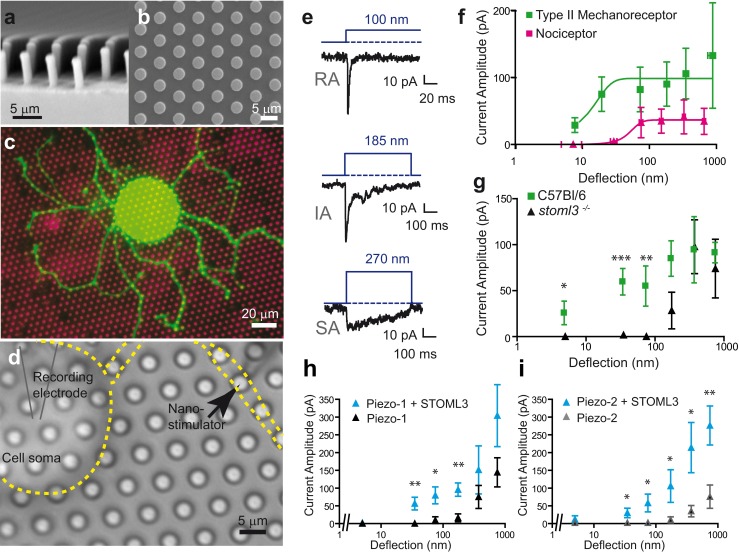
Using elastomeric pillar arrays to quantitatively measure mechanotransduction at the membrane/matrix interface. **a**, **b** Scanning electron micrographs of elastomeric pillar arrays taken perpendicular (**a**) and parallel (**b**) to the elements of the array. **c** Pillar arrays can be coated with EHS-laminin (*magenta*), and sensory neurons acutely isolated from the mouse will attach to the array and extend neurites over the tops of the pili (*green*, overexpressed LifeAct-GFP). **d** Cells can be monitored using whole-cell patch clamp, and a glass nanostimulator can be used to deflect individual pillar elements directly underneath the neurite, *bright field image*, cell outlined in *yellow*, *black arrow* indicates individual pilus being deflected. **e** Sensory neurons respond to pillar deflection with rapidly adapting (RA), intermediate-adapting (IA) and slowly adapting (SA) currents. **f** Stimulus-response curves indicate the higher sensitivity of mechanoreceptors (*n* = 8 cells) vs nociceptors (*n* = 13 cells), note a Boltzmann fit of typeII mechanoreceptor data indicates that a stimulus of 13 nm is required for half-maximal activation of mechanically gated currents in these cells. **g** The sensitivity of type II mechanoreceptors is dependent on the presence of STOML3; C57Bl/6, *n* = 8 cells; stoml3−/−, *n* = 8 cells. (**h**, **i**) In a heterologous system, HEK-293 cells, Piezo1- (**h**, *black triangles*, *n* = 9 cells) and Piezo2- (**i**, *grey triangles*, *n* = 10 cells) mediated currents are more sensitive when these channels are co-expressed with STOML3 (*cyan triangles*; Piezo1 + STOML3, *n* = 11 cells; Piezo2 + STOML3, *n* = 9 cells). Significance determined using a Student’s *t* test, *p < 0.05, **p < 0.01, ***p < 0.001. Data from [85]

**Fig. 3:**
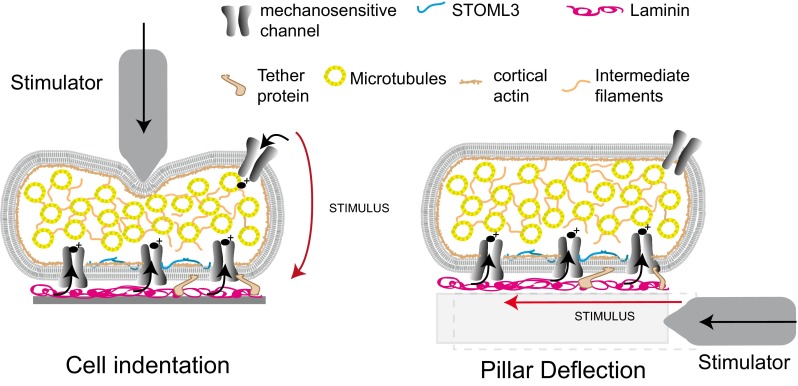
Schematic representation of indentation vs pillar deflection. When a mechanical stimulus is applied by the indentation of the soma or neurite, the stimulus is propagated via the cell to the matrix-membrane interface. In contrast, pillar deflection allows the application of fine, quantifiable stimuli directly to the membrane-matrix interface. Adapted from [85]

It is thus clear that the cell-matrix interface is critically important for the mechanosensitive currents that we are able to activate by cell body or neurite indentation in sensory neurons. Indentation techniques cannot directly activate mechanosensitive channels present in transmembrane complexes at the plasma membrane-matrix interface. In addition, the precise stimulus resulting from the indentation of the cell soma or a neurite segment with a glass probe is unknown as the size of the probe may vary from experiment to experiment, the precise moment when the probe contacts the surface of the cell is not known and the curvature and elasticity (both variable) of the impact site will modulate the stimulus as it is propagated by the cell itself to the membrane-matrix interface. We set out to design a completely new experimental approach that enables us to apply a mechanical stimulus of known magnitude directly to defined regions of the membrane-matrix interface whilst monitoring the cellular response using whole-cell patch clamp (Figs. 2 and 3). Briefly, an elastomeric pillar array was cast from a microfabricated master, with defined dimensions and material properties. To study mechanotransduction in sensory neurons, the tops of the cylindrical elements (pili) within this array are coated with laminin to promote cellular attachment and to restrict neurite outgrowth to the defined circular area. An individual pilus to which a neurite is bound can then be deflected using a nanomotor-driven stimulator, resulting in a mechanical stimulus directly at the cell-matrix interface. By applying pillar deflections with magnitudes between 10 and 1,000 nm to the plasma membrane-matrix interface, we could use whole-cell patch clamp to measure mechanosensitive currents directly activated by defined matrix deflections [85]. When applied to cultured sensory neurons, this method revealed that pillar deflection evoked RA, IA or SA currents in all cells. Importantly, the activation and inactivation kinetics of all three types of mechanosensitive currents were virtually identical to those found with neurite indentation [85]. This finding strongly suggests that the opening of channels measured after cell indentation is, at least in part, identical with those activated by matrix deflection. The pili method allows a highly defined part of membrane (10 μm^2^ in area) to be interrogated with a defined stimuli; thus, for a single neuron, we could test multiple sites. We could conclude from such experiments that mechanosensitive currents with different inactivation kinetics were often present in the same neurons. What determines the inactivation kinetics of the mechanosensitive current? It is most often assumed that channel inactivation is an intrinsic property of the channel in question. Thus, currents that inactivate with dramatically different rates may represent the activation of different channel entities. However, since the molecular nature of the channel(s) that underlie fast mechanosensitive currents is unclear, there remains the possibility that mechanical elements that are part of the mechanotransduction complex govern the rate of channel inactivation. However, several groups have described differences in the pharmacological sensitivity or ion selectivity of SA, IA and RA currents [48, 62], e.g. selective sensitivity of the SA current to block the NMB-1 peptide [30], that do suggest that currents with different inactivation properties are mediated by distinct ion channel entities.

Using pillar arrays, we could, for the first time, make accurate measurements of the magnitude of the mechanical stimulus required for current activation. Irrespective of the type of current found in sensory neurons (RA, IA or SA), we noted that nociceptors always exhibited currents with high thresholds (mostly between 200 and 1,000 nm) whereas many mechanoreceptors exhibited extraordinarily low thresholds of <50 nm. Indeed, we obtained evidence for heterogeneity within the mechanoreceptor population with one group of neurons with a distinctive action potential configuration showing the highest sensitivity. Mechanoreceptors with narrow action potentials (APs) which we designated as type II cells showed half-maximal current activation with just 13 nm of pili deflection, a molecular scale deflection corresponding to the width of half a microtubule. Interestingly, these neurons showed APs that were very reminiscent of the most sensitive cutaneous mechanoreceptor, the D-hair receptor [26, 27, 89, 94], that forms lanceolate endings around most hair follicles in the hairy skin [64, 66, 67]. Even for neurons with higher threshold deflection-sensitive currents, the mechanical stimuli needed to evoke the current were often an order of magnitude lower than the size of the indentation stimuli needed to evoke similar currents in the same cells. The pillar array system has also proved valuable for the study of proteins that modulate the sensitivity of mechanosensitive channels like STOML3, a membrane protein that appears to be a functional orthologue of the MEC-2 protein in *C. elegans* [77, 83, 96].

The mechanosensitivity of the nematode body touch receptor and mouse sensory neurons is known to be dependent on the presence of stomatin-domain-containing protein, MEC-2 in the worm and STOML3 in the mouse [49, 83, 96, 98]. Both MEC-2 and STOML3 are integral membrane proteins that insert like a hairpin into the plasma membrane leaving both N- and C-terminal peptides cytoplasmic [60]. In the absence of MEC-2, there is no detectable receptor potential in the body touch receptor neuron, and MEC-2 is a powerful positive regulator of the core mechanosensing channels MEC-4 and MEC-10 in these neurons. However, heterologously expressed MEC-4 and MEC-10 have not been described as being intrinsically mechanosensitive [11, 38]. A high-resolution X-ray crystal structure of the mouse stomatin domain from stomatin has revealed a high degree of structural similarity between mammalian and bacterial stomatin domains [9]. However, structure-function studies as well as biochemical measurements have shown that the mammalian stomatin domain from stomatin forms a stable banana-shaped dimer via C-terminal interaction domain [9]. The dimerization mode of the stomatin domain in conjunction with the membrane anchoring could conceivably produce a scaffold that could influence the way that force reaches the channels associated with STOML3. The crystal packing of stomatin dimers also suggest higher-order interaction sites on the surface of the stomatin domain that may be functionally important [9]. In the absence of STOML3, many sensory neurons lose mechanosensitive currents upon indentation of the soma or neurites. We used pillar arrays to study mechanosensitive currents gated by matrix deflection in sensory neurons lacking STOML3. Surprisingly, we found that all sensory neurons possess a deflection-gated current in *stoml3* ^−/−^ mice, but the thresholds for current deflection were dramatically elevated, up to five times larger than in wild type [85]. The effects of *Stoml3* gene deletion were most prominent in the low-threshold type II neurons, but the absence of STOML3 also led to a significant elevation of deflection thresholds in nociceptor neurons with slowly inactivating currents. The activation speed of mechanosensitive currents as well as the mechanical latency was dramatically slowed in nociceptor neurons in the absence of STOML3. These data suggest that STOML3 plays a critical role in sensitizing mechanically gated ion channels to matrix deflection. The newly discovered Piezo2 protein has been implicated as being the molecular basis of non-selective RA-type mechanosensitive currents in sensory neurons [19]; we thus asked whether the sensitivity of Piezo channels to mechanical displacement could be regulated by STOML3. We found that STOML3 can powerfully tune the sensitivity of mechanically gated Piezo1 or Piezo2 channels. In mouse neuroblastoma cells, in which the Piezo1 protein was first identified, the knockdown of *Stom3* leads to a reduction in mechanosensitive currents measured using the pili technique [85]. Thus, the interaction between STOML3 and Piezo channels appears to be necessary to maintain the sensitivity of these channels to small matrix deflections. As such, we can conclude that the scaffold protein STOML3 is necessary to maintain the sensitivity of Piezo channels to physiologically relevant membrane displacement. Indeed the profound deficits observed in the sensitivity of native mechanosensitive currents in the absence of STOML3 suggest that in vivo Piezo2 channels are physiologically regulated by the STOML3 protein. The loss of mechanosensitive current sensitivity is sufficient to effectively silence many mechanoreceptors presumably because mechanical stimuli do not drive large enough receptor potentials to initiate action potentials in these neurons. The recent finding that Piezo2 is necessary for mechanotransduction currents in Merkel cells [71, 97] raises the question whether STOML3 also plays a role in the regulation of mechanosensitivity in this cell type. STOML3 represents, to our knowledge, the first example of a protein that regulates the sensitivity of an ion channel to mechanical stimuli. The structural requirements for the modulatory activity of STOML3 appear to be quite specific and reside largely in the stomatin-domain [85]. Stomatin has a stomatin domain that, in a STOML3 backbone, is not sufficient to regulate the sensitivity of Piezo1 channels [85], and the deletion of the stomatin gene in mice only leads to moderate changes in mechanoreceptor sensitivity [73]. However, loss of stomatin together with the Deg/ENaC channel ASIC3 leads to a complete loss of sensitivity in many thinly myelinated mechanonociceptors [91].

## Concluding remarks

Many studies have shown that mechanosensing channels in sensory cells likely do not work alone, but rather within transmembrane complexes. In mammalian sensory neurons, we have shown that such complexes may anchor channels directly or indirectly to the extracellular matrix to facilitate the gating of mechanotransduction complexes in specialized sensory endings. In addition, it is clear that other proteins that can complex with and powerfully modulate mechanosensitive channels, like the Piezos, also play a critical role in regulating the sensitivity of the mechanotransducer. This regulation provides a molecular mechanism to explain the heterogeneity of mechanoreceptor sensitivity in different cutaneous receptors. The use of elastomeric pillar arrays allows the direct measurement of the activation of channels selectively at the membrane-matrix interface by mechanical deflection (Fig. 3). This will be an important tool in assessing mechanosensitive channels at membrane-matrix sites that are only partially accessible to recording with conventional techniques. A key issue question in the field is the question of whether ion channels need to be intrinsically sensitive to membrane stretch in order to serve as physiological mechanosensors. As pointed out in this review, there is no clear cut evidence that intrinsic mechanosensitivity is predictive or necessary for an ion channel protein to be a primary sensor of mechanical displacement or force.
